# Selective 5-HT_6_ Receptor Ligands (Agonist and Antagonist) Show Different Effects on Antipsychotic Drug-Induced Metabolic Dysfunctions in Rats

**DOI:** 10.3390/ph16020154

**Published:** 2023-01-20

**Authors:** Anna Partyka, Katarzyna Górecka, Joanna Gdula-Argasińska, Natalia Wilczyńska-Zawal, Magdalena Jastrzębska-Więsek, Anna Wesołowska

**Affiliations:** 1 Department of Clinical Pharmacy, Faculty of Pharmacy, Jagiellonian University Medical College, 30-688 Kraków, Poland; 2 Department of Radioligands, Faculty of Pharmacy, Jagiellonian University Medical College, 30-688 Kraków, Poland

**Keywords:** antipsychotic drugs, weight gain, metabolic disorders, 5-HT_6_ receptor, hyperglycemia, lipid profile, insulin, leptin, ghrelin, adiponectin

## Abstract

It is estimated that in patients taking antipsychotic drugs (APDs), metabolic syndrome occurs 2–3 times more often than in the general population. It manifests itself in abdominal obesity, elevated glucose concentration, and dyslipidemia. Despite the high prevalence of this disorder, only a small percentage of patients receive appropriate and effective treatment, and none of the available methods for preventing or treating APD-induced metabolic side effects is satisfactory. A promising supplement to antipsychotic therapy appears to be ligands of the serotonin 6 (5-HT_6_) receptor. The present study aimed to examine the chronic effects of the selected APDs (haloperidol, risperidone, olanzapine), administered alone and in combination with a selective 5-HT_6_ agonist (WAY-181187) or antagonist (SB-742457), on weight gain, food intake, serum lipid profile, glucose level, and a spectrum of hormones derived from adipose (leptin, adiponectin) and gastrointestinal (insulin, ghrelin) tissue in rats. SB-742457 inhibited increased weight gain and alleviated hyperglycemia induced by APDs more strongly than did WAY-181187, but also intensified dyslipidemia. WAY-181187 tended to improve the lipid profile, but increased the glucose level. The greatest benefits were obtained when WAY-181187 or SB-742457 were co-administered with haloperidol. It is difficult to assess whether the modification of the serum levels of insulin, leptin, ghrelin, and adiponectin depended on the treatment applied or other drug-independent factors; therefore, further research is needed.

## 1. Introduction

Since the early 1950s, when the first antipsychotic drug (APD), chlorpromazine, was introduced into treatment, great progress has been made in the field of psychiatric disorder pharmacotherapy. First-generation APDs (FGAs) revolutionized psychiatric care by changing its model from inpatient to outpatient. FGAs effectively reduced delusions and hallucinations (positive symptoms), but did not improve, or even had a detrimental impact, on the negative, cognitive, and affective symptoms of schizophrenia. The introduction of second-generation APDs (SGAs) broadens the range of therapeutic indications, and SGAs are currently widely used, not only for the treatment of schizophrenia or schizoaffective disorders, but also as add-on therapies in antidepressant-resistant major depressive disorder, bipolar affective disorder, or behavioral symptoms associated with dementia. Along with the discovery and introduction of newer APDs, the profile of side effects has changed. With the increasing use of SGAs, concerns about the safety and tolerability of APDs have shifted from the stigmatizing motor disorders (extrapyramidal side effects) characteristic of FGAs to the cardiometabolic disturbances affecting health, quality of life, and lifespan [1,2,3].

Chronic use of APDs, especially SGAs, is often associated with the development of metabolic abnormalities, i.e., significant weight gain, lipid disturbance, and hyperglycemia, which increase the risk of obesity, type 2 diabetes, and cardiovascular disease, and which are associated with functional impairment, reduced quality of life, and early mortality. Among the SGAs, olanzapine and clozapine are classified as compounds with the highest potential to induce weight gain and glucose dysregulation. Quetiapine, risperidone, and sertindole belong to the medium-risk group, and the compounds with the lowest metabolic risk are aripiprazole, lurasidone, and ziprasidone [4,5,6]. Despite the high prevalence of metabolic syndrome in patients treated with APDs, only a small percentage of them receive appropriate and effective treatment. Switching to an APD with lower metabolic risk, the modification of eating and physical activity habits, and the addition of supportive drugs (such as metformin, topiramat, zonisamid, or reboxetine) are the most commonly used methods to prevent and/or mitigate the metabolic side effects of APDs. However, none of these meet the expectations of both patients and physicians [7,8]. Therefore, the search for concomitant drugs is crucial for the development of better treatment options.

The substance 5-hydroxytryptamine (5-HT, serotonin) is an important factor in the regulation of appetite and body weight, and its anorectic action is well documented. Pharmacological manipulation of 5-HT_1B_, 5-HT_2C_, and recently 5-HT_6_ receptors signaling, was shown to suppress feeding and body weight in rodents [9]. The 5-HT_6_ receptors are located in multiple brain areas, including regions associated with the regulation of food intake and energy expenditure, such as the hypothalamic nuclei (the arcuate nucleus, the ventromedial nucleus, the paraventricular nucleus, and the nucleus of the solitary tract) [10,11]. In animal models, acute and chronic administration of 5-HT_6_ receptor ligands, both agonists and antagonists, resulted in decreased food intake, robust and sustained weight loss in obese animals, and reduced cardiometabolic risk (for a review, see [12,13]). Genetic evidence also supports the role of the 5-HT_6_ receptor in energy balance regulation. The short interfering RNA-mediated silencing of 5-HT_6_ gene expression induces hypophagia and weight loss [14]. Mice with a non-functional 5-HT_6_ receptor grew normally on a standard diet, but showed resistance to obesity induced by a high-fat diet [15]. All of these data support the idea that ligands of the 5-HT_6_ receptor (both agonists and antagonists) can be used as adjunct compounds to moderate the adverse metabolic effects of APDs.

The present study was designed to evaluate the combination of APD with an agonist or an antagonist of the 5-HT_6_ receptor in order to establish whether and which of the 5HT_6_ ligands would serve better in improving the metabolic safety of treatment with APDs. The study aimed to investigate the effects of chronic administration of selected APDs (haloperidol, risperidone, olanzapine), administered separately and in combination with a selective 5-HT_6_ agonist (WAY-181187) or antagonist (SB-742457), on weight gain, food intake, and biochemical parameters (serum lipid profile and glucose level) in rats. In addition, a spectrum of hormones derived from adipose (leptin, adiponectin) and gastrointestinal (insulin, ghrelin) tissue was determined under treatment conditions. Haloperidol, risperidone, and olanzapine were selected for the study because they differ in receptor profiles, represent two classes of APDs (FGAs and SGAs), and carry a different risk of metabolic complications. It is also important that APDs selected for the study differ in their affinity for the 5-HT_6_ receptor; haloperidol has no affinity (K_i_ < 5000 nM), risperidone has moderate one (K_i_ = 420 nM), and olanzapine has the highest affinity (K_i_ = 2.5 nM) [16]. WAY-181187 [17] and SB-742457 [18] were selected as tool compounds due to their high affinity and selectivity for the 5-HT_6_ receptor.

To our knowledge, the effects of the addition of 5-HT_6_ receptor ligands to APDs in the context of increased metabolic safety have not yet been investigated.

## 2. Results

### 2.1. Influence of WAY-181187 or SB-742457 on the Effects of Haloperidol, Risperidone, and Olanzapine on Cumulative Weight Gain in Rats

**Figure 1:**
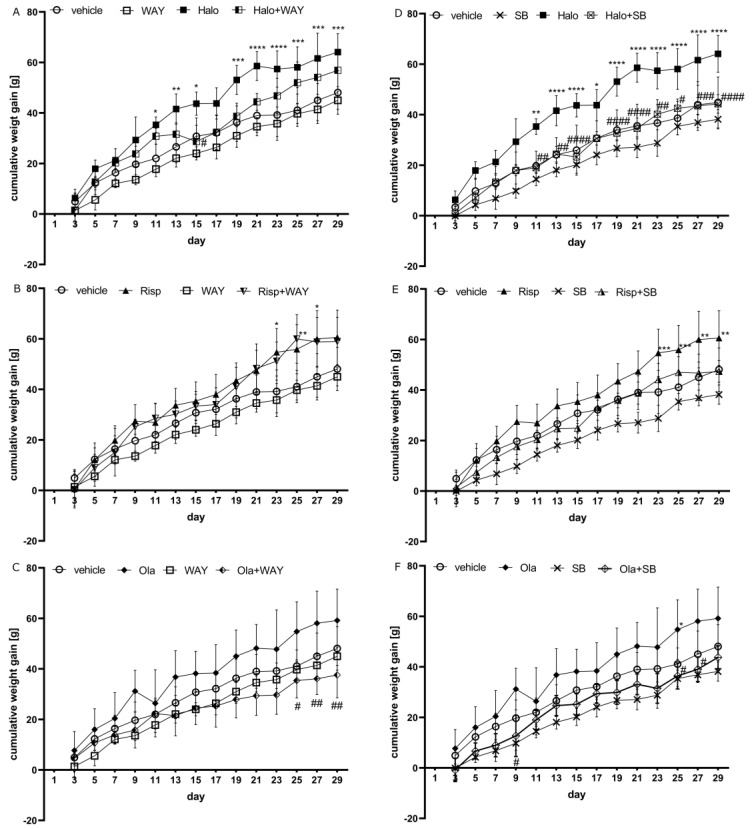
Effects of haloperidol, risperidone, olanzapine, WAY-181187 (**A**–**C**), and SB-742457 (**D**–**F**), administered alone and in combinations, on weight gain in rats. The results are presented as mean ± SEM; *n* = 9–10. * *p* < 0.05, ** *p* < 0.01, *** *p* < 0.001, **** *p* < 0.0001 versus the respective vehicle-treated group; # *p* < 0.05, ## *p* < 0.01, ### *p* < 0.001, #### *p* < 0.0001 versus the respective APD-treated group (two-way repeated measures of ANOVA followed by Bonferroni’s post hoc test). Halo, haloperidol 0.5 mg/kg; Risp, risperidone 0.5 mg/kg; Ola, olanzapine 5 mg/kg; WAY, WAY-181187 3 mg/kg; SB, SB-742457 3 mg/kg.

The cumulative weight gain over specific days in the treatment groups is shown in Figure 1. Repeated measures of ANOVA revealed a statistically significant interaction between the effects of haloperidol/WAY-181187 (F(13, 598) = 2.3609, *p* = 0.0044), olanzapine/WAY-181187 (F(13, 598) = 7.6837, *p* < 0.0001), haloperidol/SB-742457 (F(13, 598) = 4.0718, *p* < 0.0001), risperidone/SB-742457 (F(13, 598) = 2.1049, *p* = 0.0124), and olanzapine/SB-742457 (F(13, 598) = 2.0193, *p* = 0.0174) on weight gain. There was no statistically significant interaction between the effects of risperidone/WAY-181187 (F(13, 585) = 0.4827, *p* = 0.9345).

Bonferroni’s post hoc test showed that neither WAY-181187 nor SB-742457 significantly changed the cumulative weight gain in rats. All APDs tested clearly increased weight gain, but the significant changes were observed for haloperidol, from the 11th day, and risperidone, from the 23rd day, of compound administration. The effect of olanzapine did not reach statistical significance, with the exception of that noted on the 25th day. From the 25th day of the experiment, common administration of WAY-181187 and olanzapine induced a significant decrease in weight gain compared to that in the olanzapine-treated group. An insignificant decrease in weight gain was observed after administration of WAY-181187 with haloperidol, while the 5-HT_6_ receptor agonist did not change the effect of risperidone. SB-742457 reduced APD-induced weight gain, but statistically significant effects were observed after co-administration with haloperidol (from the 11th day) or olanzapine (from the 25th day).

### 2.2. Influence of WAY-181187 or SB-742457 on the Effects of Haloperidol, Risperidone, and Olanzapine on Average 48-Hour and Cumulative Food Intake in Rats

**Figure 2:**
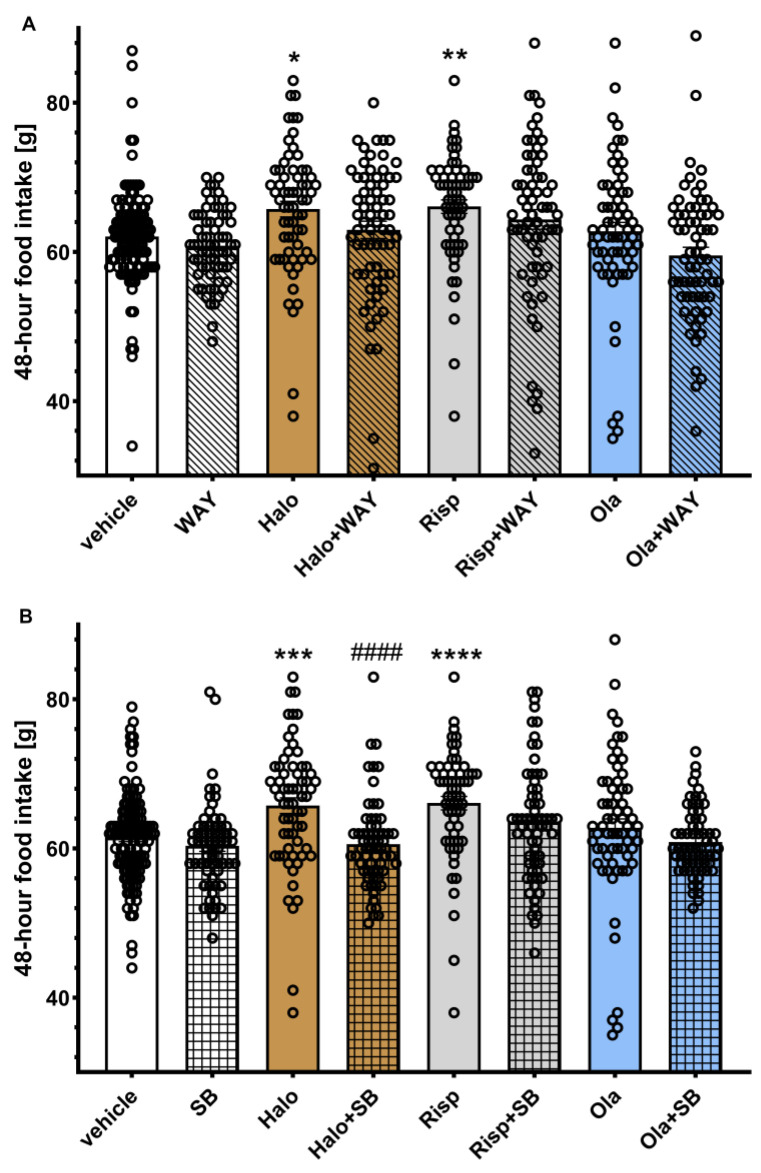
Effects of haloperidol, risperidone, olanzapine, WAY-181187 (**A**) and SB-742457 (**B**), given alone and in combinations, on average 48-hour food intake in rats. The average 48-hour intake in each group was calculated from all measurements taken throughout the experiment. The results are presented as mean ± SEM; *n* = 9–10. * *p* < 0.05, ** *p* < 0.01, *** *p* < 0.001, **** *p* < 0.0001 versus respective vehicle-treated group; #### *p* < 0.0001 versus the respective APD-treated group (two-way ANOVA followed by Bonferroni’s post hoc test). Halo, haloperidol 0.5 mg/kg; Risp, risperidone 0.5 mg/kg; Ola, olanzapine 5 mg/kg; WAY, WAY-181187 3 mg/kg; SB, SB-742457 3 mg/kg.

The average 48-hour food intake is shown in Figure 2. Two-way ANOVA revealed a statistically significant interaction between the effects of haloperidol/SB-742457 (F(1, 321) = 7.2353, *p* = 0.0075). In all other cases, the effects of the interaction between groups were not statistically significant (haloperidol/WAY-181187: F(1, 321) = 0.6621, *p* = 0.4164; risperidone/WAY-181187: F(1, 321) = 0.0714, *p* = 0.7894; olanzapine/WAY-181187: F(1, 321) = 1.0469, *p* = 0.3070; risperidone/SB-742457: F(1, 321) = 0.9026, *p* = 0.3428, olanzapine/SB-742457: F(1, 321) = 0.2616, *p* = 0.6094).

Post hoc analysis showed a statistically significant increase in 48-hour food intake in the haloperidol- and risperidone-treated groups compared to the vehicle-treated group. The addition of SB-742457, but not WAY-181187, to haloperidol significantly decreased this parameter compared to the group treated with haloperidol alone. In other cases, no significant effects of treatment on the measured parameter were observed.

**Figure 3:**
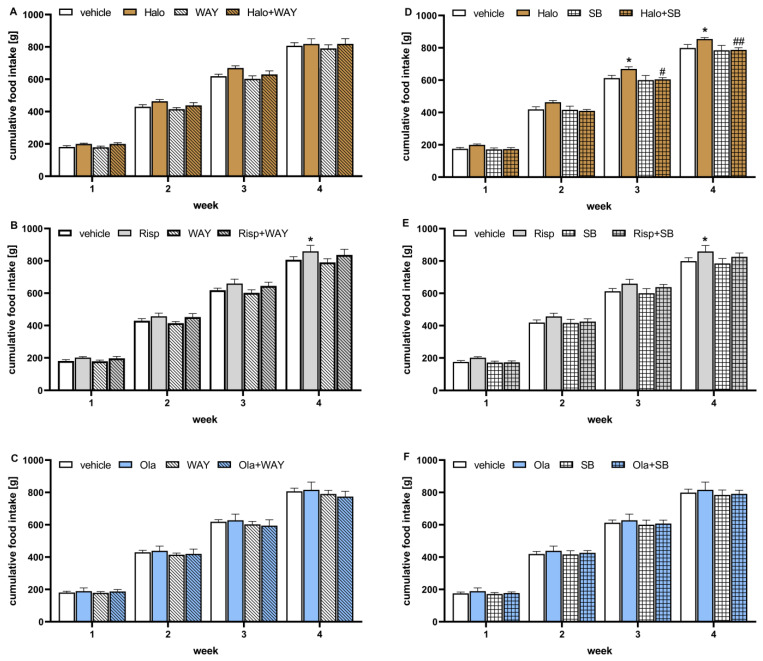
Effects of haloperidol, risperidone, olanzapine, WAY-181187 (**A**–**C**) and SB-742457 (**D**–**F**), given alone and in combinations, on cumulative food intake in rats. The results are presented as mean ± SEM; *n* = 9–10. * *p* < 0.05 versus respective vehicle-treated group; # *p* < 0.05, ## *p* < 0.01 versus the respective APD-treated group (two-way repeated measures of ANOVA followed by Bonferroni’s post hoc test). Halo, haloperidol 0.5 mg/kg; Risp, risperidone 0.5 mg/kg; Ola, olanzapine 5 mg/kg; WAY, WAY-181187 3 mg/kg; SB, SB-742457 3 mg/kg.

Cumulative food intake over weeks in the treatment groups is shown in Figure 3. Two-way repeated measures of ANOVA did not reveal a statistically significant interaction between effects of the 5-HT_6_ receptor agonist and APDs (haloperidol/WAY-181187: F(3, 63) = 1.3098, *p* = 0.2791; risperidone/WAY-181187: F(3, 63) = 0.2837, *p* = 0.8370; olanzapine/WAY-181187: F(3, 63) = 1.1825, *p* = 0.3236) nor between the 5-HT_6_ antagonist and APDs (haloperidol/SB-742457: F(3, 63) = 2.4677, *p* = 0.0702; risperidone/SB-742457: F(3, 63) = 0.6030, *p* = 0.6155; olanzapine/SB-742457: F(3, 63) = 0.0160, *p* = 0.9972).

Neither 5-HT_6_ ligands changed the cumulative food intake during the observation period. Haloperidol significantly increased cumulative food intake from the third week and risperidone from the fourth week of the experiment. Olanzapine had no influence on cumulative food intake. The addition of WAY-181187 did not change the effects of APDs, while SB-742457 significantly decreased the food intake elevated by haloperidol and had no influence on the action of the other two APDs.

### 2.3. Influence of WAY-181187 or SB-742457 on the Effects of Haloperidol, Risperidone, and Olanzapine on the Lipid Profile and Glucose Concentration in the Rat Serum

**Figure 4:**
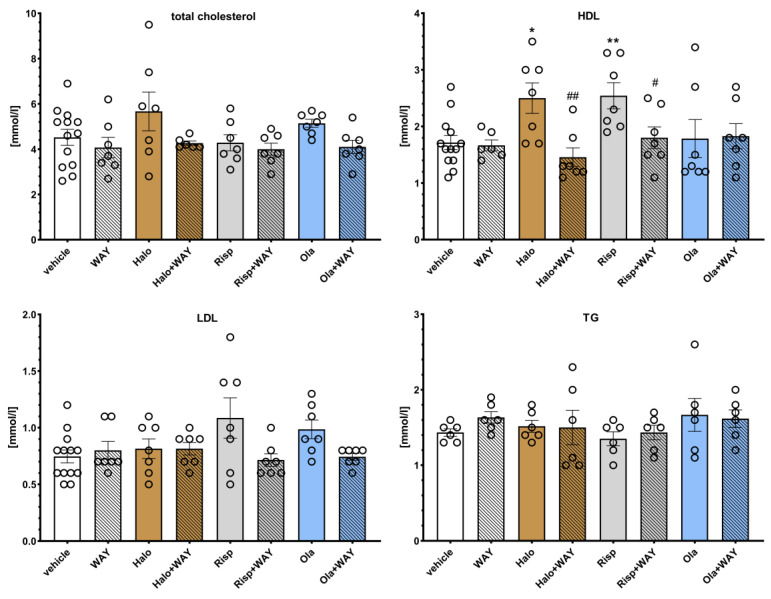
Effects of haloperidol, risperidone, olanzapine, and WAY-181187, administered alone and in combinations, on serum levels of total cholesterol, LDL, HDL, and TG in rats. The results are presented as mean ± SEM; *n* = 6–12. * *p* < 0.05, ** *p* < 0.01 versus the respective vehicle-treated group; # *p* < 0.05, ## *p* < 0.01 versus the respective APD-treated group (two-way ANOVA followed by Bonferroni’s post hoc test). Halo, haloperidol 0.5 mg/kg; Risp, risperidone 0.5 mg/kg; Ola, olanzapine 5 mg/kg; WAY, WAY-181187 3 mg/kg; LDL, low density lipoproteins; HDL, high density lipoproteins; TG, triglycerides.

The effects of WAY-181187 on APD-induced changes in the rat lipid profile are presented in Figure 4. Two-way ANOVA revealed that there was a statistically significant interaction between the effects of haloperidol/WAY-181187 on the concentration of high density lipoproteins (HDL) (F(1, 29) = 7.2513, *p* = 0.0117); haloperidol/WAY-181187 (F(1, 30) = 4.6700, *p* = 0.0388) and olanzapine/WAY-181187 (F(1, 30) = 4.8977, *p* = 0.0340) at the level of low density lipoproteins (LDL). There was no statistically significant interaction between the effects of haloperidol/WAY-181187 (F(1, 29) = 0.8147, *p* = 0.3742), risperidone/WAY-181187 (F(1, 30) = 0.0480, *p* = 0.8281), and olanzapine/WAY-181187 (F(1, 30) = 0.6026, *p* = 0.4437) on total cholesterol concentration; between the effects of haloperidol/WAY-181187 (F(1, 30) = 0.0695, *p* = 0.7939) on LDL concentration; the effects of risperidone/WAY-181187 (F(1, 29) = 3.8243, *p* = 0.0602) and olanzapine/WAY-181187 (F(1, 29) = 0.0795, *p* = 0.7800) on HDL concentration; and between the effects of haloperidol/WAY-181187 (F(1, 20) = 0.6242, *p* = 0.4388), risperidone/WAY-181187 (F(1, 20) = 0.3621, *p* = 0.5541) and olanzapine/WAY-181187 (F(1, 20) = 0.75881, *p* = 0.3940) on triglycerides (TG) concentration.

WAY-181187 alone did not significantly change the lipid profile in rats. Haloperidol and risperidone significantly increased HDL and risperidone levels, and an increase in LDL concentration was observed at the statistical significance limit after administration of risperidone (*p* = 0.0662) and olanzapine (*p* = 0.0729). The addition of WAY-181187 to haloperidol resulted in a significant decrease in HDL level and an insignificant decrease in total cholesterol level. Co-administration of risperidone and WAY-181187 caused a significant decrease in HDL concentration and an insignificant decrease in LDL concentration. The addition of WAY-181187 to olanzapine insignificantly lowered the level of LDL compared to that in the olanzapine-treated group. In other cases, no significant effects of the applied treatments on the measured parameters were observed.

**Figure 5:**
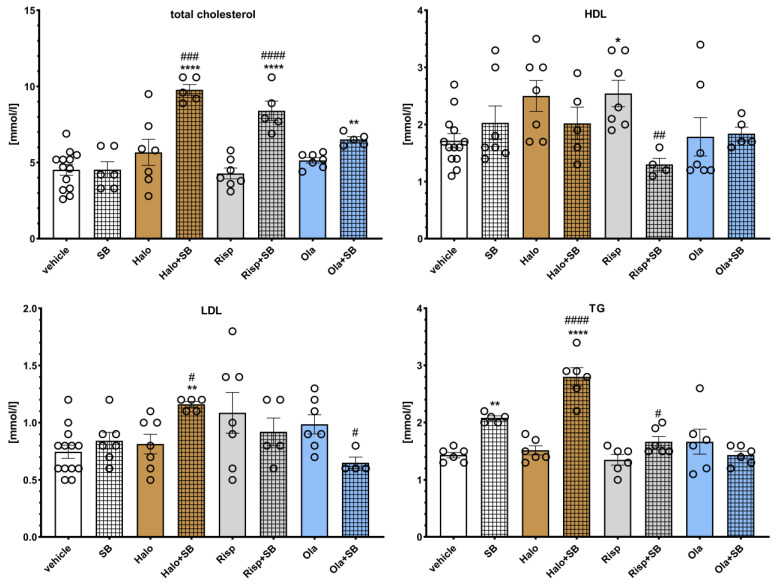
Effects of haloperidol, risperidone, olanzapine, and SB-742457, administered alone and in combinations, on serum levels of total cholesterol, LDL, HDL, and TG in rats. The results are presented as mean ± SEM; *n* = 6–12. * *p* < 0.05, ** *p* < 0.01, **** *p* < 0.0001 versus respective vehicle-treated group; # *p* < 0.05, ## *p* < 0.01, ### *p* < 0.001, #### *p* < 0.0001 versus the respective APD-treated group (two-way ANOVA followed by Bonferroni’s post hoc test). Halo, haloperidol 0.5 mg/kg; Risp, risperidone 0.5 mg/kg; Ola, olanzapine 5 mg/kg; SB, SB-742457 3 mg/kg; LDL, low density lipoproteins; HDL, high density lipoproteins; TG, triglycerides.

The effects of SB-742457 on APD-induced changes in the rat lipid profile are presented in Figure 5. Two-way ANOVA revealed that there was a statistically significant interaction between the effects of haloperidol/SB-742457 on total cholesterol concentration (F(1,27) = 12.839, *p* = 0.0013) and TG level (F(1, 19) = 9.0145, *p* = 0.0073); risperidone/SB-742457 on total cholesterol concentration (F(1, 27) = 18.724, *p* = 0.0002), HDL concentration (F(1, 27) = 12.890, *p* = 0.0013) and TG level (F(1, 19) = 5.2448, *p* = 0.0336), as well as olanzapine/SB-742457 on LDL (F(1, 27) = 8.4552, *p* = 0.0072) and TG (F(1, 19) = 12.001, *p* = 0.0026) levels. There was no significant interaction between the effects of olanzapine/SB-742457 (F(1, 27) = 2.9396, *p* = 0.0979) on total cholesterol concentration; between the effects of haloperidol/SB-742457 (F(1, 28) = 3.8313, *p* = 0.0603) and risperidone/SB-742457 (F(1, 19) = 5.2448, *p* = 0.0336) on LDL concentration; or between the effects of haloperidol/SB-742457 (F(1, 28) = 2.8607, *p* = 0.1019) and olanzapine/SB-742457 (F(1, 28) = 0.2953, *p* = 0.5912) on HDL concentration.

When administered alone, SB-742457 did not have an effect on the concentration of the lipid profile components, except for on TG, which was significantly elevated. Risperidone significantly increased HDL level. An increase in HDL concentration was also observed at the statistical significance limit after haloperidol administration (*p* = 0.0815). Administration of risperidone and olanzapine resulted in an elevation of LDL level to the statistical significance limit (*p* = 0.0925 and *p* = 0.0891, respectively). The 5-HT_6_ antagonist added to haloperidol caused a significant increase in total cholesterol, LDL, and TG concentrations compared to the results for the haloperidol- and vehicle-treated groups. The combined administration of SB-742457 and risperidone significantly increased the total cholesterol level and decreased the HDL concentration compared to the risperidone- and vehicle-treated groups, and increased TG concentration compared to the risperidone-treated group. Concomitant administration of SB-742457 and olanzapine resulted in a significant decrease in LDL levels compared to the olanzapine-treated group and caused a significant elevation in the total cholesterol concentration compared to the vehicle-treated group. In other cases, no significant effects of the applied treatment on the measured parameters were observed.

**Figure 6:**
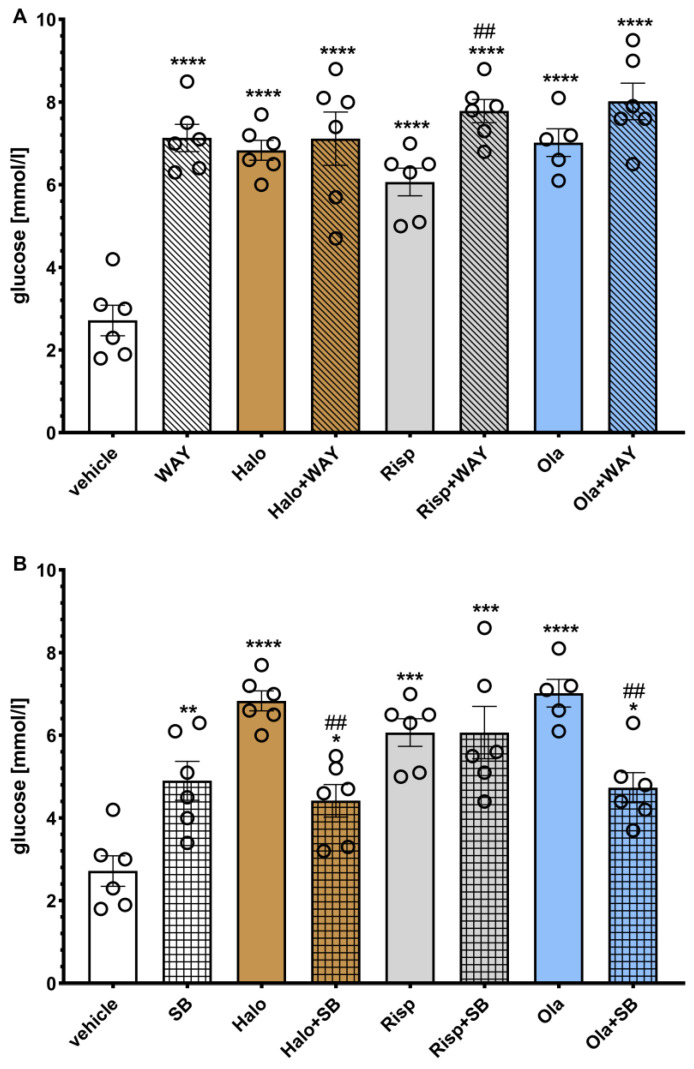
Effects of haloperidol, risperidone, olanzapine, WAY-181187 (**A**) and SB-742457 (**B**), administered alone and in combinations, on the glucose level in rat serum. The results are presented as mean ± SEM; *n* = 5–6. * *p* < 0.05, ** *p* < 0.01, *** *p* < 0.001, **** *p* < 0.0001 versus respective vehicle-treated group; ## *p* < 0.01 versus the respective APD-treated group (two-way ANOVA followed by Bonferroni’s post hoc test). Halo, haloperidol 0.5 mg/kg; Risp, risperidone 0.5 mg/kg; Ola, olanzapine 5 mg/kg; WAY, WAY-18187 3 mg/kg; SB, SB-742457 3 mg/kg.

The effects of WAY-181187 or SB-742457 on ADP-induced changes in the rat serum glucose levels are presented in Figure 6. A two-way ANOVA revealed that there was a statistically significant interaction between the effects of haloperidol/WAY-181187 (F(1, 20) = 23.753, *p* = 0.0001), risperidone/WAY-181187 (F(1, 20) = 16.695, *p* = 0.0006), olanzapine/WAY-181187 (F(1, 19) = 20.071, *p* = 0.0003), haloperidol/SB-742457 (F(1, 20) = 36.827, *p* < 0.0001), risperidone/SB-742457 (F(1, 20) = 5.3723, *p* = 0.0312), and olanzapine/SB-742457 (F(1, 19) = 32.104, *p* < 0.0001).

Both 5-HT_6_ ligands and all three APDs, administered separately and in combination, significantly increased the glucose concentration compared to the vehicle-treated group. The addition of WAY-181187 to haloperidol or olanzapine did not significantly change the glucose level compared to a group treated with the respective APD, while common administration with risperidone resulted in a significant increase in the glucose concentration compared to the group treated with risperidone. SB-742457 significantly decreased the measured parameter when combined with haloperidol or olanzapine, but not risperidone, compared to the respective APD-treated group.

### 2.4. Influence of WAY-181187 or SB-742457 on the Effects of Haloperidol, Risperidone, and Olanzapine on Insulin, Leptin, Ghrelin, and Adiponectin Levels in the Rat Serum

**Figure 7:**
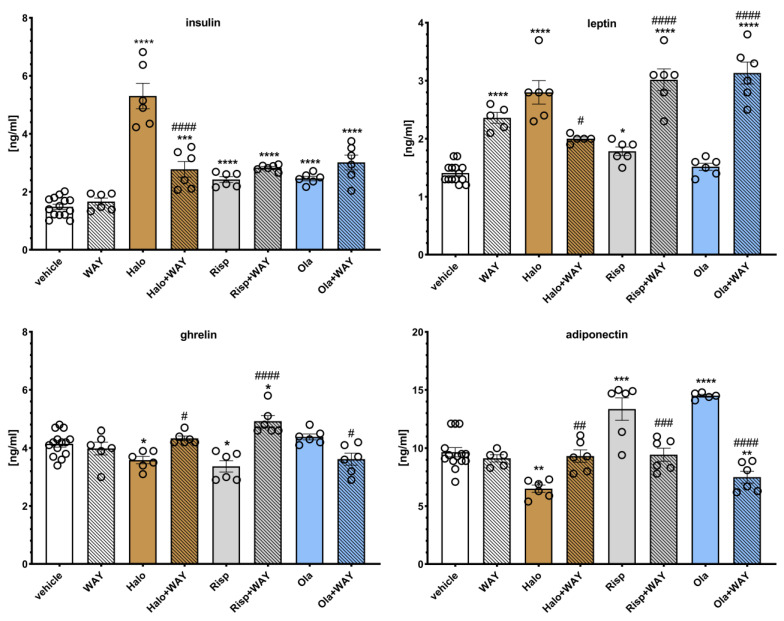
Effects of haloperidol, risperidone, olanzapine, and WAY-181187, administered alone and in combinations, on the serum levels of insulin, ghrelin, leptin, and adiponectin in rats. The results are presented as mean ± SEM; *n* = 6–12. * *p* < 0.05, ** *p* < 0.01, *** *p* < 0.001, **** *p* < 0.0001 versus the respective vehicle-treated group; # *p* < 0.05, ## *p* < 0.01, ### *p* < 0.001, #### *p* < 0.0001 versus respective APD-treated group (two-way ANOVA followed by Bonferroni’s post hoc test). Halo, haloperidol 0.5 mg/kg; Risp, risperidone 0.5 mg/kg; Ola, olanzapine 5 mg/kg; WAY, WAY-181187 3 mg/kg.

The effects of WAY-181187 on APD-induced changes in hormone levels in rat serum are presented in Figure 7. Two-way ANOVA revealed that there was a statistically significant interaction between the effects of haloperidol/WAY-181187 (F(1, 28) = 36.822, *p* < 0.0001) on insulin concentration; between the effects of haloperidol/WAY-181187 (F(1, 26) = 71.397, *p* < 0.0001), olanzapine/WAY-181187 (F(1, 27) = 12.084, *p* = 0.0017) on leptin level; between the effects of haloperidol/WAY-181187 (F(1, 28) = 9.5359, *p* = 0.0045) and risperidone/WAY-181187 (F(1, 28) = 23.570, *p* < 0.0001) on ghrelin concentration, and between the effects of haloperidol/WAY-181187 (F(1, 27) = 11.447, *p* = 0.0022), risperidone/WAY-181187 (F(1, 27) = 7.4480, *p* = 0.0110), and olanzapine/WAY-181187 (F(1, 26) = 42.976, *p* < 0.0001) on adiponectin concentration. There was no significant interaction between the effects of risperidone/WAY-181187 (F(1, 28) = 1.2049, *p* = 0.2817) and olanzapine/WAY-181187 (F(1, 28) = 1.7244, *p* = 0.1998) on insulin concentration; between the effects of risperidone/WAY-181187 (F(1, 27) = 2.0641, *p* = 0.1623) on leptin level, and between the effects of olanzapine/WAY-181187 (F(1, 28) = 3.1119, *p* = 0.0886)) on ghrelin concentration.

A significant increase in leptin concentration and no significant effects on the serum levels of insulin, ghrelin, and adiponectin were observed when WAY-181187 was administered alone. Haloperidol significantly increased the serum levels of insulin and leptin and decreased the concentrations of ghrelin and adiponectin. Risperidone elevated insulin, leptin, and adiponectin levels and decreased the ghrelin concentration. Olanzapine increased insulin and adiponectin concentrations and had no effect on the leptin and ghrelin levels. The addition of WAY-181187 to haloperidol resulted in a significant decrease in insulin and leptin levels and an increase in ghrelin and adiponectin concentrations compared to the group treated with haloperidol. The co-administration of risperidone and WAY-181187 caused a significant increase in leptin and ghrelin concentrations, lowered the level of adiponectin, and had no effect on the insulin concentration compared to the risperidone-treated group. The addition of WAY-181187 to olanzapine significantly lowered the ghrelin and adiponectin levels, increased the serum leptin concentration, and did not significantly modify the insulin level compared to those in the group treated with olanzapine.

**Figure 8:**
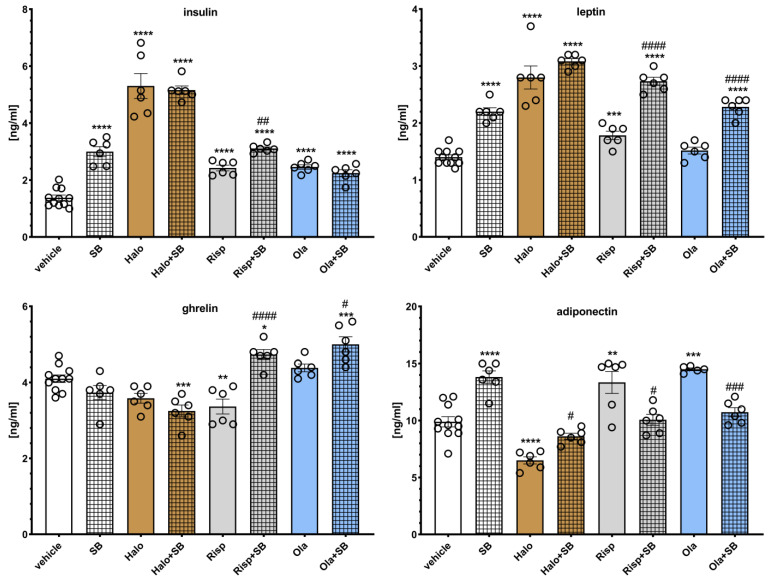
Effects of haloperidol, risperidone, olanzapine, and SB-742457, administered alone and in combinations, on the serum levels of insulin, ghrelin, leptin, and adiponectin in rats. The results are presented as mean ± SEM; *n* = 6–11. * *p* < 0.05, ** *p* < 0.01, *** *p* < 0.001, **** *p* < 0.0001 versus the respective vehicle-treated group; # *p* < 0.05, ## *p* < 0.01, ### *p* < 0.001, #### *p* < 0.0001 versus respective APD-treated group (two-way ANOVA followed by Bonferroni’s post hoc test). Halo, haloperidol 0.5 mg/kg; Risp, risperidone 0.5 mg/kg; Ola, olanzapine 5 mg/kg; SB, SB-742457 3 mg/kg.

The effects of SB-742457 on the APD-induced changes in hormone levels in the rat serum are presented in Figure 8. Two-way ANOVA revealed that there was a statistically significant interaction between the effects of haloperidol/SB-742457 (F(1, 25) = 15.849, *p* = 0.0005), risperidone/SB-742457 (F(1, 25) = 15.699, *p* = 0.0006), and olanzapine/SB-742457 (F(1, 25) = 55.656, *p* < 0.0001) on insulin concentration; between the effects of haloperidol/SB-742457 (F(1, 25) = 6.5380, *p* = 0.0170) on the level of leptin; between the effects of risperidone/SB-742457 (F(1, 25) = 32.796, *p* < 0.0001) and olanzapine/SB-742457 (F(1, 25) = 11.591, *p* = 0.0022) on ghrelin concentration, and between the effects of risperidone/SB-742457 (F(1, 25) = 33.320, *p* < 0.0001) and olanzapine/SB-742457 (F(1, 24) = 63.194, *p* < 0.0001) on adiponectin concentration. There was no significant interaction between the effects of risperidone/SB-742457 (F(1, 25) = 0.8312, *p* = 0.3706) and olanzapine/SB-742457 (F(1, 25) = 0.1240, *p* = 0.7277) on the level of leptin, between the effects of haloperidol/SB-742457 (F(1, 25) = 0.0277, *p* = 0.8692) on ghrelin concentration, and between the effects of haloperidol/SB-742457 (F(1, 25) = 3.8122, *p* = 0.0622) on the level of adiponectin.

When administered alone, SB-742457 significantly increased serum levels of insulin, leptin, and adiponectin, having no effect on ghrelin concentration. Haloperidol significantly increased the serum levels of insulin and leptin and decreased ghrelin (no statistical significance) and adiponectin concentrations. Risperidone elevated insulin, leptin, and adiponectin levels and decreased ghrelin concentration. Olanzapine increased insulin and adiponectin concentrations and had no effect on leptin and ghrelin levels. SB-742457 did not significantly influence the effects of haloperidol on insulin, leptin, and ghrelin concentration and increased the level of adiponectin compared to that in the group treated with haloperidol. The 5-HT_6_ antagonist significantly modified risperidone-induced changes in measured hormone levels, increasing the concentration of insulin, leptin, and ghrelin, and lowering adiponectin level in the serum of rats, compared to the risperidone-treated group. The addition of SB-742457 to olanzapine resulted in an elevation in serum leptin and ghrelin levels and a decrease in the concentration of adiponectin.

## 3. Discussion

The present study is the first to demonstrate the effects of adding a selective 5-HT_6_ ligand (agonist or antagonist) to APDs on metabolic disorders caused by repeated administration (for 28 days) of different APDs in rats. The data obtained revealed that the 5-HT_6_ receptor antagonist, SB-742457, showed a stronger lowering effect than the 5-HT_6_ receptor agonist, WAY-181187, on increased weight gain and food intake induced by haloperidol administration. The effects of risperidone and olanzapine were modified by the 5-HT_6_ receptor ligands in a similar and equal way. The opposite influence of the addition of WAY-181187 and SB-742457 to APDs was observed on the lipid profile and glucose levels. The effect of the 5-HT_6_ antagonist was more pronounced and generally led to the intensification of lipid disorders and the reduction in APD-induced hyperglycemia, while the addition of the 5-HT_6_ agonist contributed to a slight improvement in the lipid profile and the aggravation of glycemic disorders. In terms of the effect of 5-HT_6_ receptor ligands on hormones involved in the regulation of weight gain and appetite, only the addition of WAY-181187 to haloperidol attenuated post-APD changes in their serum concentrations. Both WAY-181187 and SB-742457 contributed to the reduction in the increased level of adiponectin after risperidone or olanzapine administration, but increased the effect of these drugs on leptin and, to a lesser extent, insulin and ghrelin concentrations (Table 1).

Animal models of APD-induced metabolic side effects are widely described in the scientific literature. While researchers tend to agree on the usefulness of preclinical studies in understanding the mechanisms involved in the metabolic abnormalities induced by APDs, the metabolic changes observed in animals vary significantly depending on the species and strain of rodents, the APDs used, and their dosage, and may be inconsistent even with the same drug [19]. Accordingly, in our study, the APDs tested induced various metabolic changes to a different extent. Surprisingly, the strongest and most consistent metabolic disturbances were observed after 4 weeks of administration of haloperidol, then risperidone, and finally, olanzapine. Haloperidol caused a significant increase in food intake accompanied by an increase in weight gain, hyperglycemia, and a slight increase in total cholesterol level. A significant elevation in serum insulin and leptin levels and a decrease in ghrelin and adiponectin concentrations were also observed. Similar effects were observed after risperidone administration, except for a weaker impact on food intake and increased serum adiponectin concentration were noted, while olanzapine induced hyperglycemia, increased insulin and adiponectin levels, and showed a statistically insignificant impact on weight gain and lipid profile (Table 1). In all cases, hyperglycemia was accompanied by an increase in insulin concentration, which indirectly suggests the presence of insulin resistance, although confirmation of this phenomenon would require additional tests (for example, an oral glucose tolerance test).

In clinical practice, haloperidol is not classified as an APD that causes severe undesirable metabolic effects; however, patients gain weight during its use [4,6,20,21]. Moreover, in animal studies, haloperidol caused metabolic changes such as weight gain, hyperinsulinemia, hyperglycemia, or hypercholesterolemia; however, its effects are generally weaker than those of SGAs [22,23,24,25,26]. Clinically, risperidone is classified as an APD with a medium metabolic risk [6], inducing moderate weight gain and changes in the lipid profile and glucose level [4]. In animal studies, risperidone increased body weight [23,27,28], serum levels of total cholesterol and TG, induced hyperinsulinemia, and altered glucose metabolism [29,30]. Olanzapine has a high potential to cause weight gain, hyperglycemia, and hypercholesterolemia in humans [4,21] and is most widely used in preclinical studies on the undesirable metabolic action of APDs [19,31]. Contrary to expectations, in our experiment, olanzapine only induced statistically significant hyperglycemia and hyperinsulinemia and insignificantly increased weight gain and concentrations of total cholesterol, LDL, and TG. The lack of statistically significant differences was due to the relatively high interindividual variability in response to the drug and the high SEM associated with it. It is likely that temporary sedation occurred for several hours after olanzapine administration (our own unpublished observations), resulting in more sensitive rats with lower food intake and contributed to a greater variability of the results obtained. However, the discrepancies between the literature and our data do not seem to differ significantly [19].

The underlying mechanism of APD-induced metabolic changes is complex and includes antagonism of histamine H_1_, serotonin 5-HT_2C/2A_, dopamine D_2/3_, muscarinic M_3_, and adrenergic α_1_ receptors. This is still an unelucidated issue. One of the research areas concerns the role of peptide hormones such as leptin, ghrelin, and adiponectin. Leptin is secreted by adipocytes and participates in the central regulation of appetite, reducing it, and the peripheral regulation of metabolic activity, increasing the effects of insulin and improving the utilization of fatty acids in skeletal muscle [32]. In patients treated with olanzapine, marked weight gain was associated with an increase in serum leptin levels, while those treated with haloperidol did not show substantial weight gain or elevated leptin concentrations. For risperidone, clinical trials observed moderate changes in weight and leptin levels [3,33]. Animal data are less consistent. In female rats, olanzapine induced weight gain, but serum leptin levels did not differ between treatment groups [34], while in the study with clozapine, both body weight and leptin levels were increased [35]. As expected, in our study, a significant increase in serum leptin concentration was observed in the haloperidol or risperidone treated group, in which there was also a greater weight gain. Olanzapine, which caused only a slight increase in body weight compared to the control group, did not change the level of leptin.

Unlike leptin, ghrelin induces hunger by activating orexigenic hypothalamic neurocircuits, resulting in increased food intake and stimulated adipogenesis. Ghrelin is produced mainly in the endocrinal cells of the gastric glands. The level of ghrelin increases before meals, and food suppresses its release [36]. Studies in patients taking APDs are completely inconsistent and show an increase, a decrease, and no change in serum ghrelin levels [33,37]. The tendency to increase the level of ghrelin (effect at the statistical significance limit) was reported in rats fed clozapine, but not haloperidol [22]. Another study showed a significant elevation in serum ghrelin levels after treatment with long-acting injectable haloperidol or risperidone, but not with olanzapine, in rats [38]. On the contrary, in the investigations by Weston-Green et al., olanzapine significantly increased total ghrelin [39]. Our results revealed that haloperidol and risperidone decreased the serum level of ghrelin, while olanzapine did not affect it. Such contradictory reports make it difficult to discuss the results and make it impossible to draw definitive conclusions about the effect of APDs on ghrelin levels and its association with the metabolic changes induced by these drugs.

Adiponectin is an adipose-specific protein. In humans, its level is negatively correlated with body weight and insulin levels [40]. As with ghrelin, data from patients treated with APDs are conflicting; in most studies, no changes in serum adiponectin levels were reported, but there was also evidence for increased and decreased levels [3,33,41]. Similar results were obtained from animal studies that demonstrated no impact of olanzapine [42] or haloperidol [22], an increase in serum adiponectin level after olanzapine [34,43] or clozapine [22,44] treatment, as well as an increase in adiponectin mRNA in the visceral fat of rats treated with risperidone [45]. In the present study, the serum level of adiponectin was lowered only in the haloperidol-treated rats; in animals treated with risperidone or olanzapine, the peptide concentration was increased, while the insulin level was elevated in all APD-treated groups. However, we determined the total adiponectin in serum, and the studies suggest a closer correlation between high molecular weight adiponectin and insulin sensitivity [46].

Scientific data indicate that 5-HT_6_ antagonism rather than agonism is important for the anti-obesity effect. The 5-HT_6_ receptor antagonists (PRX-07034, Ro 04-6790, BVT 5182, SB 271046, and idalopirdine) significantly reduced food intake and body weight in obese rodents and promote satiety in normal-weight rats [12,13,47,48]. Similar effects showed that the partial agonists of the 5-HT_6_ receptor, E-6837, caused hypophagia in non-obese and diet-induced obese rats [49], and EMD 386088 induced weight loss in rats fed a high-fat diet and reduced food intake in models of excessive eating and obesity induced by the high-fat diet [50]. Our results showed that WAY-181187 and SB-742457 administered at a dose of 3 mg/kg (that is, a dose at which both ligands improved memory deficits in rats in our previous studies [51]) did not influence weight gain, food intake, or lipid profile, except for increasing the serum TG level by SB-742457. Unfortunately, both 5-HT_6_ ligands caused severe hyperglycemia. It seems that in the case of the 5-HT_6_ receptor antagonist, this effect may be the result of reduced insulin sensitivity because at the same time, a significant increase in the serum level of this hormone was observed. Both 5-HT_6_ receptor ligands increased serum leptin concentration. Although the lack of effect on body weight and food intake is not contradictory to the majority of literature data showing that such effects are revealed primarily in various models of obesity or after acute administration of compounds to non-obese rats, the increase in TG, glucose, and leptin concentrations contradicts the published data [13]. The 5-HT_6_ receptor ligands reduced adiposity and visceral fat stores, which was reflected in decreased serum leptin levels. Improvements in glycemic control were reflected in a reduction in serum insulin concentration, without a change in glucose levels. The 5-HT_6_ receptor ligands generally did not have an effect on TG levels, although two studies showed a decrease in serum TG concentrations [13,49]. The reason for these discrepancies may result from methodological differences. First of all, the published data pertain to obese rats, while our studies were carried out in animals of normal weight.

The results obtained suggest that the addition of a 5-HT_6_ receptor ligand, both an agonist and an antagonist, to an APD may beneficially modify the metabolic consequences of the chronic use of these drugs. However, this effect is not uniform and depends on both the type of ligand and the neuroleptic. SB-742457 appears to have a greater inhibitory effect on APD-induced weight gain. This finding is supported by the vast majority of reports in the literature indicating that 5-HT_6_ antagonism, rather than agonism, is responsible for weight loss. However, it should be noted that, when combined with olanzapine, both ligands had a similar effect. This phenomenon is difficult to explain, especially since olanzapine has the highest affinity for the 5-HT_6_ receptor among the studied APDs and is the antagonist of this receptor. The reduction in weight gain was related to a decrease in food intake, although statistically significant differences were obtained only when SB-742457 was administered in combination with haloperidol. The hypophagic effect of the selective 5-HT_6_ antagonist, SB-399885, was shown to be associated with increased activation of neurons in two hypothalamic nuclei, the paraventricular nucleus and the nucleus of the solitary tract, which promotes anorectic behavior [52]. The hypophagic effect of 5-HT_6_ ligands is explained by the blocking or attenuating of the action of 5-HT on γ-aminobutyric acid (GABA) interneurons in the hypothalamus, which results in reduced GABA release and a subsequent increase in the release of anorexic α-melanocyte-stimulating hormone (α-MSH) [53] and/or the inhibition of hunger-promoting agouti-related peptide (AgRP)/GABA neurons in the arcuate nucleus of the hypothalamus [52]. The favorable action of SB-742457 on weight gain and food intake was accompanied by a significant aggravation of lipid parameters, such as increased total cholesterol, LDL, and TG, relative to the respective APD-treated group. WAY-181187 did not significantly change these parameters, but showed a tendency to improve the lipid profile. Interestingly, the effect of 5-HT_6_ ligands on APD-induced hyperglycemia was the complete opposite; WAY-181187 exacerbated glycemic disturbances, while SB-742457 decreased glucose concentrations, however, not at the level of the vehicle-treated group. The least consistent are the data on the effect of the 5-HT_6_ ligands on APD-induced changes in serum hormone levels. WAY-181187 and SB-742457 acted unidirectionally or inversely, and no regularities were found in these effects in the context of hormone or APD. For this reason, it is difficult to find connections between the effect of treatment on body weight and metabolic parameters and levels of insulin, leptin, ghrelin, or adiponectin. The lack of data from the scientific reports on the effect of the combined administration of APDs with 5-HT_6_ receptor ligands does not allow any conclusions to be drawn. The results obtained from the haloperidol and WAY-181187 interaction experiment seem to be the most consistent. Haloperidol given alone increased weight gain, total cholesterol, and glucose levels, which were accompanied by elevated insulin concentration, decreased adiponectin, and increased leptin levels. When WAY-181187 was administered along with haloperidol, the tendency to decrease weight gain and total cholesterol was observed, and the insulin concentration was reduced, without changes in the glucose level. As expected, these changes were accompanied by an increase in adiponectin and a decrease in leptin serum concentrations.

The present study has some limitations. First, this research is part of a larger project aimed at assessing the potential benefits that can result from the combined use of APDs and selective 5-HT_6_ receptor ligands on metabolic side effects, cognitive impairments, and depressive- and/or anxiety-like symptoms, and the same doses were used to evaluate all these aspects. This could have been the reason for not obtaining the full spectrum of metabolic disorders induced by olanzapine. Second, for the same reason (the need to perform other animal studies and collect tissues immediately after their completion), the rats were not fasted prior to blood sampling. Therefore, the results of glucose, TG, and some hormones can be difficult to interpret. However, it should be emphasized that all experimental groups were maintained under the same conditions, and the results of the drug-treated groups were compared with those of the appropriate vehicle-treated group. Third, the research presented is preliminary and aims only to estimate whether the common administration of a selective 5-HT_6_ receptor agonist/antagonist with the selected APD will help mitigate its adverse metabolic effects. It would also be interesting to investigate how this pharmacological manipulation affects the daily activity and metabolic rate of animals. Finally, it is not known whether the results obtained are general and can be extrapolated to other APDs and/or other 5-HT_6_ ligands. In further research, it will be necessary to take into account all of the above issues.

## 4. Materials and Methods

### 4.1. Animals

The experiment was carried out in female Wistar rats purchased from the Animal House at the Faculty of Pharmacy, Jagiellonian University Medical College, Kraków, Poland. At the time of arrival, the animals weighed 160–180 g.

During the experiment, the rats were kept in an environmentally controlled laboratory room under the following conditions: temperature 22 ± 2 °C, humidity 55 ± 10%, 12 h light/dark cycles (light on at 7:00 a.m. and off at 7:00 p.m.). The rats were housed in pairs in standard plastic cages (L × W × H) 378 × 217 × 180 mm. Wood blocks, paper tubes, and strips were used to enrich the environment. The rats had free access to food (standard laboratory pellets) and tap water.

A total of 140 rats were used in the study, and each treatment group consisted of 10 randomly selected animals. Due to the large number of animals and limited laboratory space, the experiment was carried out in three turns: the first included 4 treatment groups: vehicle (1% Tween 80), haloperidol, risperidone, and olanzapine; the second consisted of 5 treatment groups: vehicle (1% Tween 80), WAY-181187, haloperidol + WAY-181187, risperidone + WAY-181187, and olanzapine + WAY-181187; and the third consisted of 5 treatment groups: vehicle (1% Tween 80), SB-742457, haloperidol + SB-742457, risperidone + SB-742457, and olanzapine + SB-742457. One animal died during the administration of the compounds; therefore, one experimental group (i.e., risperidone + WAY-181187-treated group) eventually consisted of only 9 animals. 24 hours after the last drug administration, trained personnel sacrificed the rats by dislocating the cervical spinal cord.

The research was carried out according to EU Directive 2010/63/EU and Polish legal regulations (DzU 2015 item 266, as amended). All animal procedures were approved by the II Local Ethics Commission at the Institute of Pharmacology PAS in Kraków (approval No. 107/2016). The 3R rule was implemented in the study. A procedure for early, humane termination of the experiment was developed in the event of significant deterioration of the animal’s health. The procedure was intended to be initiated when at least two of the following symptoms occurred: convulsions, respiratory disturbance, movement disorder, immobility, lack of water and/or food intake, muscle relaxation, or lack of touch response.

### 4.2. Drugs and Treatment

Haloperidol (TargetMol, Boston, MA, USA), risperidone (TargetMol), olanzapine (TargetMol), WAY-181187 (oxalate; Tocris Bioscience, Bristol, UK), and SB-742457 (TargetMol) were used in the experiment. Doses of APDs (haloperidol 0.5 mg/kg, risperidone 0.5 mg/kg, and olanzapine 5 mg/kg) and 5-HT_6_ ligands (WAY-181187 3 mg/kg and SB-742457 3 mg/kg) were selected for the experiments, based on literature review and our previous studies which presented their separate and combined behavioral effects [54]. The compounds were suspended in a 1% solution of Tween 80 (Sigma Aldrich, St. Louis, MO, USA) immediately before administration and injected intraperitoneally (ip) in a volume of 2 mL/kg. The compounds were dispensed to the rats once daily between 10:00 and 11:00 a.m. for 28 days. The last injection was given 24 h before sacrifice. The control rats received 1% Tween 80, on the same dosing regimen.

### 4.3. Measurement of Body Weight and Food Intake

The body weight of each rat and the food intake per cage were measured alternately, every other day, to the nearest 0.1 g using an electronic scale. Measurements were performed before drug administration.

### 4.4. Sample Collection

The animals were sacrificed by decapitation, and the blood from the trunk was collected in plastic tubes. The tubes were stored for 20 min at room temperature to allow the serum to clot. The samples were centrifuged at 300× *g* at 20 °C for 20 min. The serum was collected and stored at −80 °C for further biochemical analysis.

### 4.5. Biochemical Analysis

The serum concentrations of the biochemical parameters were measured spectrophotometrically using commercially available rat enzyme-linked immunosorbent assay (ELISA) kits (Bioassay Technology Laboratory, Shanghai, China, for total cholesterol, LDL, HDL, TG, and ghrelin; Mediagnost, Reutlingen, Germany, for adiponectin and leptin; Crystal Chem, Elk Grove Village, IL, USA, for insulin and glucose), according to the manufacturers’ instructions. Absorbance was measured using the Omega Star microplate reader (BMG LABTECH, Ortenberg, Germany).

### 4.6. Statistical Analysis

Statistical analysis was performed using the Statistica 13 program. Data were presented as mean ± standard error of mean (SEM). The two-way repeated measures of analysis of variance (ANOVA), with drug 1 (5-HT_6_ ligand) and drug 2 (APD) as between-subject factors and time as the repeated measures factor, was used to evaluate the effects of body weight gain and food intake. The results of biochemical analysis were examined by two-way ANOVA, with the between-subject factors drug 1 (5-HT_6_ ligand) and drug 2 (APD). The post hoc Bonferroni’s comparison test was used to compare between groups. Differences between groups were considered significant when the *p*-value was <0.05.

## 5. Conclusions

In conclusion, the results obtained provide us with an unambiguous answer regarding whether the addition of a selective 5-HT_6_ agonist or antagonist will bring more benefits concerning post-APD metabolic disorders. The greatest benefits were obtained when the 5-HT_6_ ligand was co-administered with haloperidol, which, unlike risperidone (K_i_ = 420 nM) and olanzapine (K_i_ = 2.5 nM), has no affinity for the 5-HT_6_ receptor (K_i_ > 5000 nM) [16]. WAY-181187 normalized haloperidol-induced changes in the serum levels of peptides regulating appetite and metabolism activity and, to a lesser extent, decreased weight gain and food intake, while SB-742457 strongly reduced weight gain and food intake and was less likely to modify hormonal changes. Generally, SB-742457 more strongly inhibited increased weight gain and alleviated the hyperglycemia caused by APDs, but it should be noted that it also intensified dyslipidemia. On the other hand, WAY-181187 tended to improve the lipid profile, but increased the glucose level. It is also difficult to assess whether the modification of the serum levels of insulin, leptin, ghrelin, and adiponectin depended on the treatment applied or other drug-independent factors (for example: weight gain, daily locomotor activity, adipose tissue content); therefore, further research is needed.

## Figures and Tables

**Table 1:** Effects of separate and combined administration of tested APDs and 5-HT_6_ receptor ligands on measured parameters.

Parameter	Halo	Halo + WAY	Halo + SB	Risp	Risp + WAY	Risp + SB	Ola	Ola + WAY	Ola + SB
Weight gain	**↑**	↘	**↓**	**↑**	**-**	↘	↗	**↓**	**↓**
48 h food intake	**↑**	↘	**↓**	**↑**	↘	↘	-	↘	↘
Total food intake	**↑**	-	**↓**	↗	-	-	-	↘	↘
Total cholesterol	↗	↘	**↑**	-	-	**↑**	↗	↘	↗
LDL	-	-	**↑**	↗	↘	**↑**	↗	↘	**↓**
HDL	**↑**	**↓**	↘	**↑**	**↓**	**↓**	-	-	-
TG	-	-	**↑**	-	-	**↑**	↗	-	↘
Glucose	**↑**	-	**↓**	**↑**	**↑**	-	**↑**	↗	**↓**
Insulin	**↑**	**↓**	-	**↑**	-	**↑**	**↑**	↗	-
Leptin	**↑**	**↓**	↗	**↑**	**↑**	**↑**	-	**↑**	**↑**
Ghrelin	**↓**	**↑**	↘	**↓**	**↑**	**↑**	-	**↓**	**↑**
Adiponectin	**↓**	**↑**	**↑**	**↑**	**↓**	**↓**	**↑**	**↓**	**↓**

When APDs were administered separately, their effects were compared to those of the vehicle-treated group. When APDs were administered in combination with 5-HT_6_ ligands, the effects of this combination were compared to the respective APD-treated group; **↑**/**↓**—statistically significant increase/decrease in the measured parameter, ↗/↘—a tendency of action (non-significant effect), “-”—no effect; HALO—haloperidol 0.5 mg/kg; RISP—risperidone 0.5 mg/kg; OLA—Olanzapine 5 mg/kg; WAY—WAY-181187 3 mg/kg; SB—SB-74245714. 3 mg/kg; LDL—low density lipoprotein; HDL—high density lipoprotein; TG—triglycerides.

## Data Availability

Data is contained within the article.
